# Removal Performance and Mechanistic Insights into As(V) Transport in Natural Manganese Minerals

**DOI:** 10.3390/toxics14040340

**Published:** 2026-04-17

**Authors:** Zhicheng Zhao, Huimei Shan, Song Wei, Zheying Li, Qingsheng Li

**Affiliations:** 1 Guangxi Key Laboratory of Environmental Pollution Control Theory and Technology, Guilin University of Technology, Guilin 541006, China; zzcheng1261@163.com (Z.Z.); weis@glut.edu.cn (S.W.); 15837571133@163.com (Z.L.); m17513334889@163.com (Q.L.); 2 Collaborative Innovation Center for Water Pollution Control and Water Safety in Karst Area, Guilin University of Technology, Guilin 541006, China

**Keywords:** natural manganese minerals, arsenic, adsorption and migration, Two-Site Kinetic Adsorption Model (TSKAM)

## Abstract

Arsenic contamination in polymetallic mining areas is closely linked to surrounding iron-rich manganese minerals. However, conclusive evidence remains limited regarding the retention and migration process of As(V) in naturally manganese-rich manganese ores (especially those with different manganese/iron mass ratios) under dynamic flow conditions. This study investigated As(V) adsorption and transport by four natural manganese minerals (FM1–FM4) through batch/column experiments, characterization, and numerical modeling. Their Mn/Fe mass ratios were 22.7 for FM1, 4.2 for FM2, 3.7 for FM3, and 16.4 for FM4. Batch experiments showed that As(V) adsorption on FM1–FM3 was better described by the Freundlich model, indicating heterogeneous adsorption behavior. Under the tested experimental conditions, the apparent Langmuir *q*_m_ values of these minerals decreased from 0.066 to 0.015 mmol·g^−1^ with decreasing Mn/Fe ratio. However, As(V) adsorption on FM4, which had the lowest Mn and Fe contents, followed the Langmuir model (*q*_m_ = 0.012 mmol·g^−1^), suggesting monolayer adsorption. Column experiments demonstrated rapid As(V) retention for all minerals. In the time domain, increasing the flow rate from 0.5 to 2.0 mL·min^−1^ generally advanced breakthrough and shortened the desorption tail, although the breakthrough behavior expressed in pore-volume coordinates was not strictly monotonic for all minerals. The Two-Site Kinetic Attachment Model (TSKAM) successfully simulated these dynamics (R^2^ > 0.90, RMSE < 0.05), revealing adsorption controlled by fast and slow kinetic sites, with slow-site contributions diminishing at higher flow rates. Characterization results indicated that adsorbed arsenic on FM1 remained mainly as As(V) and was immobilized primarily through surface complexation involving surface hydroxyl and Fe/Mn–O groups. XRD and SEM-EDS suggested the participation of Fe/Mn-bearing phases, while XPS on FM1 showed pronounced changes in Mn surface species during adsorption. Therefore, As(V) removal by these natural manganese minerals is a coupled physicochemical process influenced by both mineral properties, including Mn/Fe ratio, specific surface area, pore structure, pH_PZC_, and Mn surface-state changes, and hydrodynamic conditions in the polymetallic mining areas.

## 1. Introduction

Arsenic (As), a highly toxic metalloid commonly found in groundwater, is linked to various health issues, including cancer, cardiovascular diseases, and neurological damage upon chronic exposure. High arsenic concentrations threaten groundwater in over 70 countries, with particularly severe cases in South Asia, China, and parts of Southeast Asia [1,2]. The mobilization and transport of arsenic in groundwater are governed by both natural geochemical processes—such as the weathering and dissolution of arsenic-bearing minerals, and changes in redox conditions and pH—and anthropogenic activities like mining and agricultural fertilization [3,4,5]. Consequently, mitigating and controlling arsenic pollution is a critical research priority in hydrology and environmental geochemistry [6].

In natural systems, manganese (Mn) minerals are recognized as key phases controlling arsenic behavior due to their strong oxidative capacity and high sorption affinity [7,8]. Moreover, the adsorption and redox mechanisms of arsenic species on mineral surfaces have been extensively investigated, particularly in studies of Fe/Mn oxides and other model mineral systems [9,10,11]. Naturally occurring Mn minerals are often iron-rich, and this unique Mn-Fe binary characteristic allows them to mitigate arsenic transport through multiple mechanisms: Mn oxides can strongly oxidize As(III) [12] and form stable inner-sphere complexes with As(V) [13], while Fe oxides tend to immobilize arsenic via surface adsorption and co-precipitation [14,15]. The Mn/Fe ratio in different natural manganese minerals directly influences the density and type of reactive surface sites, thereby determining the dominant mechanism of arsenic immobilization; minerals with higher Mn/Fe ratios generally exhibit superior sorption performance for arsenic [16,17,18,19].

In solute transport studies, the one-dimensional Advection-Dispersion Equation (ADE) is frequently employed as a foundational model [20,21]. However, ADE primarily describes hydrodynamic transport in the liquid phase and cannot fully capture nonequilibrium retention processes at the solid–liquid interface [22,23]. The two-site kinetic attachment framework used in this study originates from colloid-transport modeling rather than from classical dissolved-solute transport models [24,25]. In its original form, this framework describes reversible and irreversible or capacity-limited attachment processes in porous media [24,25]. Here, we adopt the Two-Site Kinetic Attachment Model (TSKAM) as a phenomenological extension of ADE to represent heterogeneous As retention during column transport. A recent study further demonstrated the applicability of this framework to arsenic transport by using TSKAM to simulate the coupled transport and adsorption–desorption behavior of As in heterogeneous karst aquifers [26]. In the present study, Br^−^ was used as a conservative tracer and interpreted with the ADE to constrain hydrodynamic dispersion, whereas TSKAM was applied to simulate the reactive transport of As(V). Nevertheless, its application to natural manganese mineral-arsenic systems remains limited. In particular, a systematic integration of mineralogical characterization, batch/column experimental results, and TSKAM modeling is lacking, which constrains the explanatory and predictive power of the model for arsenic transport in real-world groundwater systems.

Guangxi, known as the “hometown of non-ferrous metals,” in China, has a long history of mining that has brought economic benefits but also led to severe heavy metal and metalloid pollution, with arsenic contamination in water bodies being the most critical [27]. Surveys indicate significant spatial and temporal variability in arsenic pollution in the polymetallic mining areas in the region, a characteristic potentially linked to the influence of surrounding iron-manganese minerals; however, the underlying mechanisms remain insufficiently resolved [28]. While extensive research has focused on contaminant adsorption by synthetic Mn-Fe oxides or single-mineral systems [29], comparatively less is known about how natural manganese minerals, with their complex composition and multi-scale structure, regulate As transport under dynamic groundwater flow conditions. Existing studies often rely on batch equilibrium experiments [30], which, while valuable for revealing sorption capacities and interfacial reactions, cannot fully capture dynamic transport and breakthrough processes in groundwater. Although TSKAM has been applied in column studies of several contaminants. its use in natural manganese mineral-arsenic systems remains limited, particularly for comparative studies involving natural minerals with different Mn/Fe ratios that integrate breakthrough curves, model fitting, and mineralogical characterization [31,32].

Given these research gaps, this study investigates the adsorption and transport of As(V) using four natural manganese minerals with different Mn/Fe ratios collected from a mining area in Hechi City, Guangxi. The removal efficiency and transport retardation of these minerals are compared through batch and column experiments. The dominant mechanisms are elucidated using complementary characterization techniques, including X-ray diffraction (XRD), scanning electron microscopy with energy-dispersive X-ray spectroscopy (SEM-EDS), Fourier-transform infrared spectroscopy (FTIR), and X-ray photoelectron spectroscopy (XPS). Meanwhile, Br^−^ tracer experiments interpreted with ADE are used to constrain hydrodynamic parameters, and TSKAM is employed to simulate the breakthrough behavior of reactive As(V) through parameter inversion and model validation. This integrated approach enhances our understanding of how natural manganese minerals regulate arsenic transport and provides a quantitative basis for the in situ remediation of arsenic-contaminated groundwater.

## 2. Materials and Methods

### 2.1. Experimental Materials

All reagents, including sodium arsenate heptahydrate (Na_2_HAsO_4_·7H_2_O), sodium chloride (NaCl), and potassium bromide (KBr), were of analytical grade. Sodium hydroxide (NaOH), hydrochloric acid (HCl), concentrated nitric acid (HNO_3_), hydrofluoric acid (HF), and perchloric acid (HClO_4_) were of guaranteed grade. All reagents were purchased from Xilong Scientific Co., Ltd. (Guangzhou, China) An As(V) stock solution (13.35 mmol·L^−1^) was prepared using analytical grade Na_2_HAsO_4_·7H_2_O. All aqueous solutions were prepared with ultrapure water (18.25 MΩ·cm). Quartz sand was sequentially soaked in 0.01 M HNO_3_, 0.01 M NaOH, and 30% H_2_O_2_ for 24 h each, then thoroughly rinsed with deionized water until a stable electrical conductivity (approx. 35 μS·cm^−1^) was achieved to remove surface impurities. Four natural iron-bearing manganese minerals, collected as rock fragments from a mining area in Hechi City, Guangxi, were used in this study. The iron and manganese contents of the different minerals were determined using acid digestion. Based on their Mn/Fe mass ratios, the minerals were designated as FM1 (22.7), FM2 (4.2), FM3 (3.7), and FM4 (16.4). The rock fragments were washed with water, air-dried naturally, and then ground to a 40-mesh (375 μm) particle size using a ball mill. The minerals used in the batch experiments were further sieved to a particle size of approximately 150 μm, whereas those used in the column experiments were prepared to a particle size of approximately 375 μm. The powdered samples were stored in sealed containers until use.

### 2.2. Experimental Methods

#### 2.2.1. Batch Experiments

To investigate the interaction behavior of As(V) with natural manganese minerals under ambient laboratory atmosphere without deliberate exclusion of O_2_/CO_2_, all experiments in this study, including the column experiments, were carried out under ambient laboratory atmosphere, and oxygen and carbon dioxide were not deliberately excluded.

**Adsorption Isotherms:** For each mineral, 1.0 ± 0.001 g of powdered sample (particle size approximately 150 μm) was accurately weighed into 50 mL centrifuge tubes. Subsequently, 20 mL of As(V) solution at concentrations of 0.13, 0.27, 0.67, 1.34, 2.67, and 6.67 mmol·L^−1^ was added to each tube. The tubes were sealed and placed in a constant temperature oscillator (180r·min^−1^) at 25 ± 1 °C and pH 7 ± 0.2 for 24 h to improve liquid-solid contact under constant-temperature conditions. All experiments were performed in triplicate. After oscillation, 10mL of the supernatant was carefully aspirated, filtered through a 0.45 μm membrane, and analyzed for residual arsenic concentration.

**Adsorption Kinetics:** 1.0 ± 0.001 g of each mineral powder (particle size approximately 150 μm) was weighed into 50 mL centrifuge tubes and mixed with 20 mL of 2.67 mmol·L^−1^ As(V) solution. The tubes were sealed and agitated in a constant temperature oscillator (180r·min^−1^) at 25 ± 1 °C and pH 7 ± 0.2 for predetermined time intervals (10 min, 30 min, 1 h, 2 h, 4 h, 6 h, 8 h, 10 h, 12 h, 16 h, 20 h, and 24 h) to improve liquid–solid contact under constant-temperature conditions. Each time point was tested in triplicate. After each interval, 10mL of the supernatant was carefully collected with a pipette to avoid disturbing the mineral precipitate, filtered through a 0.45 μm membrane, and analyzed for arsenic concentration. An independent centrifuge tube was used for each sampling time point rather than repeated sampling from the same tube; therefore, the decrease in sample volume did not affect the adsorption results.

**Effect of initial pH:** 1.0 ± 0.001 g of mineral powder (particle size approximately 150 μm) was added to 50 mL centrifuge tubes containing 20 mL of 2.67 mmol·L^−1^ As(V) solution. The initial pH of the suspensions was precisely adjusted to 1, 3, 5, 7, 9, and 11 using 0.1 mol·L^−1^ HCl or NaOH. The sealed tubes were then oscillated (180r·min^−1^) at 25 ± 1 °C for 24 h to improve liquid-solid contact under constant-temperature conditions. All experiments were performed in triplicate. After oscillation, 10mL of the supernatant was carefully collected, filtered (0.45 μm), and analyzed for arsenic concentration.

**Adsorption–Desorption:** For the adsorption phase, 1.0 ± 0.001 g of mineral powder (particle size approximately 150 μm) was mixed with 20 mL of 2.67 mmol·L^−1^ As(V) solution in 50 mL centrifuge tubes. The pH was adjusted to 7, and the tubes were oscillated (180r·min^−1^) at 25 ± 1 °C for 24 h to improve liquid–solid contact under constant-temperature conditions. All experiments were performed in triplicate. After oscillation, 10mL of the supernatant was collected, filtered (0.45 μm), and analyzed for arsenic. Following adsorption, the supernatant was carefully decanted, and the mineral solid was retained. The solid was rinsed with a saturated NaCl solution to remove any residual solution and impurities. Subsequently, 20 mL of 0.1 mol·L^−1^ NaOH solution was added to the tubes, since previous studies have shown that 0.1 M NaOH can provide effective regeneration while minimizing structural degradation. The mixture was then oscillated for another 24 h at 25 ± 1 °C to improve liquid-solid contact under constant-temperature conditions. After this desorption phase, the suspension was centrifuged, and the supernatant was filtered and analyzed for arsenic concentration.

#### 2.2.2. Column Experiments

**Figure 1:**
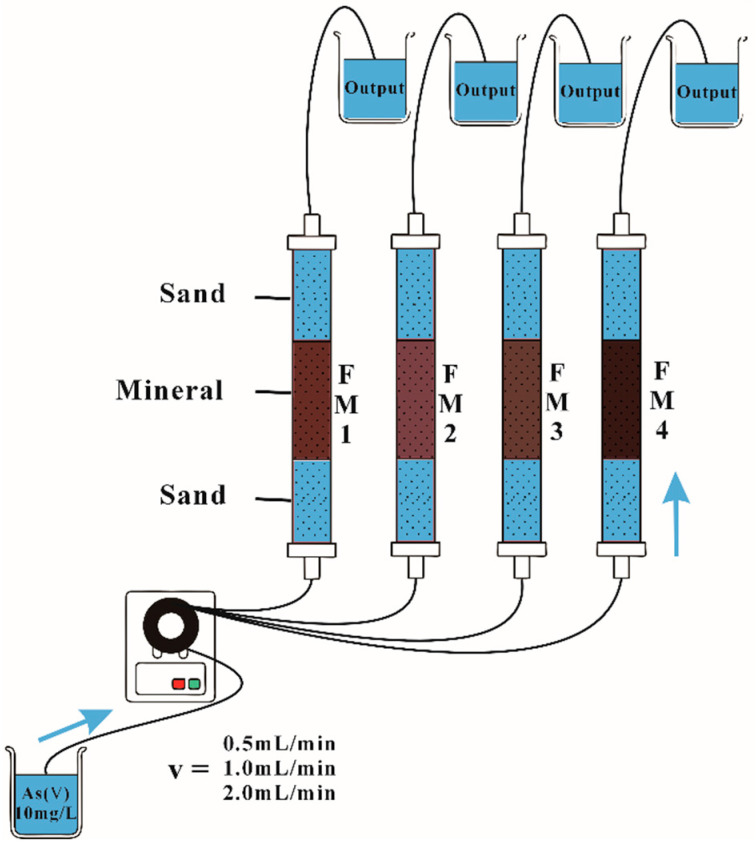
Schematic diagram of the experimental device. The arrows indicate the direction of solution flow through the column system.

To systematically evaluate As(V) adsorption onto natural manganese minerals with varying Mn/Fe ratios, a fixed-bed glass column experiment system was designed and constructed (Figure 1). Glass columns (1.6 cm inner diameter, 15 cm length) were packed from bottom to top as follows: a 5 cm supporting layer of quartz sand (particle size approximately 375 μm), a 5 cm layer of natural iron-manganese mineral (particle size approximately 375 μm, pre-rinsed and dried at 60 °C), and a 5 cm covering layer of quartz sand. The column ends were sealed with polyethylene caps equipped with inlet/outlet ports and fitted with 300-mesh (aperture approximately 48 μm) screens to prevent solid loss. Columns were wet-packed, and the wall was gently tapped after each layer to ensure uniform packing. A total of 12 breakthrough experiments were conducted using columns packed with the four minerals (Mn/Fe ratios were 22.7 for FM1, 4.2 for FM2, 3.7 for FM3, and 16.4 for FM4), each at three flow rates (0.5, 1.0, and 2.0 mL·min^−1^). Initially, a 0.01 M CaCl_2_ background solution was pumped upward through the column at a stable flow rate for saturation. After gravity drainage, the column was allowed to equilibrate for 2–3 days. For the adsorption phase, an As(V) solution (0.13 mmol·L^−1^, pH 7.0) was introduced upward into the column using a multichannel peristaltic pump (DT100-1F, Baoding Lead Fluid Technology Co., Ltd., Baoding, China). When the effluent As concentration (*C*_e_) relative to the influent concentration (*C*_0_) reached *C*_e_/*C*_0_ ≥ 0.9, the influent was switched to deionized water for the desorption phase. The experiment was terminated when *C*_e_/*C*_0_ ≤ 0.1. The inflow and outflow rates were kept constant. Effluent samples were collected after filtration through 0.45 μm membranes and analyzed for Fe, Mn, As, pH, and electrical conductivity. Before the column experiments, pH was monitored to ensure that the influent solution met the target experimental condition. During adsorption, only limited routine pH checks were carried out on collected effluent samples. For each collected sample, the analytical determination was performed in duplicate to reduce analytical deviation and improve data reliability. After each experiment, the column packing material was retrieved, dried, and ground for post-reaction characterization.

#### 2.2.3. Sample Analysis and Characterization

Solution pH and electrical conductivity (EC) before and after reactions were measured using a portable multi-parameter digital analyzer (Hach-HQ30d, Hach Company, Loveland, CO, USA). In the batch pH-effect experiment, the pH values reported in this study refer to the initial pH adjusted before adsorption. Total dissolved arsenic concentrations were determined using an atomic fluorescence spectrometer (AFS 933/SA-20, Beijing Jitian Instruments Co., Ltd., Beijing, China; detection limit ≤ 0.01 μg·L^−1^). In this study, dissolved As, Fe, and Mn concentrations are reported on an elemental molar basis (mmol·L^−1^). For elemental analysis of the pristine minerals, a 100 mg sample of homogenized mineral powder (passed through a 0.149 mm nylon sieve) was digested in a 30 mL polytetrafluoroethylene crucible with a mixed acid solution (HNO_3_:HF = 1:7 *v*/*v*) on a hot plate. HClO_4_ was then added, and heating continued until white fumes ceased and the residue appeared light-colored. The residue was dissolved in HNO_3_, and the solution was diluted to volume. Elemental concentrations in the digestate were determined using inductively coupled plasma optical emission spectrometry (ICP-OES). The elemental compositions of the four natural manganese minerals are presented in Table 1.

Specific surface area (SSA) was calculated using the Brunauer–Emmett–Teller (BET) method. The specific surface areas of FM1–FM4 are given in Table 2. The point of zero charge (pH_PZC_) was determined using a Zeta potential analyzer (Zetasizer Nano ZS90, Malvern Panalytical Ltd., Malvern, UK). Before pH_PZC_ measurement, the mineral samples were sieved to below 100 μm to obtain a finer fraction for analysis. Mineral phase composition was analyzed by X-ray diffraction (XRD, XPert3 Powder, Malvern Panalytical, B.V., Almelo, The Netherlands). Functional groups on the solid samples were characterized by Fourier-transform infrared spectroscopy (FTIR, iS 10, Thermo Fisher Scientific, Waltham, MA, USA) using the KBr pellet method. Surface morphology and elemental distribution were examined using field emission scanning electron microscopy coupled with energy-dispersive X-ray spectroscopy (SEM-EDS, JSM-7900F, JEOL Ltd., Tokyo, Japan). Surface elemental composition and chemical bonding states of the solid samples before and after reactions were analyzed by X-ray photoelectron spectroscopy (XPS, ESCALAB 250Xi, Thermo Fisher Scientific Inc., Waltham, MA, USA). Before solid-phase characterization, all reacted solid samples were sieved to satisfy the analytical requirements of the corresponding tests.

#### 2.2.4. Models and Calculations

In the batch experiments, the arsenic adsorption capacity was calculated using Equation (1), and the arsenic removal efficiency (*η*) by the natural manganese minerals was determined using Equation (2):

(1) $$q_{e}=\frac{(C_{0}-C_{e})V}{m}$$

(2) $$\eta =\frac{C_{0}-C_{e}}{C_{0}}\times 100\%$$

where *q*_e_ is the adsorption capacity (mmol·g^−1^), *C*_0_ and *C*_e_ are the initial and equilibrium concentrations of As(V) in the solution (mmol·L^−1^, expressed as dissolved As), *V* is the volume of the As(V) solution (L), and m is the mass of the mineral (g).

Batch equilibrium data were fitted to the Langmuir and the Freundlich isotherm models to describe As(V) adsorption onto the minerals [33]. The Langmuir model [34,35] assumes uniform adsorption sites with identical affinity, leading to monolayer adsorption. The Freundlich model [36] is an empirical equation used to describe heterogeneous adsorption systems. For both isotherm models, the parameters were obtained by nonlinear regression of the original, non-linearized *q*_e_-*C*_e_ data, and the goodness of fit was evaluated using R^2^. The models are expressed as Equations (3) and (4), respectively:

(3) $$q_{e}=\frac{bC_{e}q_{m}}{1+bC_{e}}$$

(4) $$q_{e}=K_{f}C_{e}^{\frac{1}{n}}$$

where *K*_f_ is the Freundlich constant, with units of (mmol·g^−1^) (L·mmol^−1^)^1/*n*^, related to adsorption capacity, *n* is the Freundlich heterogeneity factor, *b* is the Langmuir constant (L·mmol^−1^) related to adsorption energy, and *q*_m_ is the maximum Langmuir adsorption capacity (mmol·g^−1^).

Kinetic data were fitted to pseudo-first-order [37] and pseudo-second-order [38,39] models, as shown in Equations (5) and (6):

(5) $$ln(q_{e}-q_{t})=lnq_{e}-K_{1}t$$

(6) $$\frac{t}{q_{t}}=\frac{1}{K_{2}q_{e}^{2}}+\frac{t}{q_{e}}$$

where *q*_t_ and *q*_e_ are the amounts of As adsorbed (mmol·g^−1^) at time *t* and at equilibrium, respectively; *K*_1_ is the pseudo-first-order rate constant (min^−1^); and *K*_2_ is the pseudo-second-order rate constant (g·mmol^−1^·min^−1^).

To facilitate comparison of breakthrough curves among columns packed with different minerals and operated at different flow rates, the transport data were expressed as a function of pore volume (PV). PV was calculated as:

(7) $$PV=\frac{Q_{t}}{V_{P}}$$

where *Q* is the influent flow rate (mL·min^−1^), t is the elapsed time (min), and $V_{p}$ is the pore volume of the packed column (mL). The calculated $V_{p}$ values for the FM1, FM2, FM3, and FM4 columns were 5.31, 5.09, 3.75, and 4.52 mL, respectively. Accordingly, the time corresponding to 1 PV varied with flow rate for each mineral column.

Because Br^−^ interacts only weakly with Mn/Fe oxides, it was used in this study as a conservative tracer to characterize hydrodynamic transport in the packed columns rather than adsorption behavior. Br^−^ has been widely used in column and soil transport studies for constraining dispersion-related parameters, although its conservative behavior should be interpreted cautiously in reactive topsoils [40,41]. The Br^−^ breakthrough curves were therefore analyzed using the one-dimensional advection-dispersion equation (ADE), and the fitted hydrodynamic dispersion coefficient D was subsequently fixed in the reactive transport simulations of As(V) [42]. The governing equation for ADE is:

(8) $$\frac{\partial \theta C}{\partial t}=\frac{\partial }{\partial x}(\theta D\frac{\partial C}{\partial x})-v\frac{\partial C}{\partial x}$$

where *θ* is the volumetric water content; *C* is the solute concentration in the liquid phase (mg·L^−1^); *D* is the dispersion coefficient (cm^2^·min^−1^); *v* is the Darcy velocity (cm·min^−1^).

Arsenic transport in the saturated sand columns is controlled by adsorption–desorption kinetics. Among process-based models, the Two-Site Kinetic Attachment Model (TSKAM), which accounts for both reversible and irreversible adsorption/desorption, has been successfully used to describe the transport of complex solutes in porous media [43,44]. Therefore, TSKAM was employed in this study to simulate the transport of As in the saturated sand columns. The governing equations for TSKAM are:

(9) $$\frac{\partial \theta C}{\partial t}+\rho_{b}\frac{\partial S_{1}}{\partial t}+\rho_{b}\frac{\partial S_{2}}{\partial t}=\frac{\partial }{\partial x}(\theta D\frac{\partial C}{\partial x})-v\frac{\partial C}{\partial x}$$

(10) $$\rho_{b}\frac{\partial S_{1}}{\partial t}=\theta k_{1}C-\rho_{b}k_{1d}S_{1}$$

(11) $$\rho_{b}\frac{\partial S_{2}}{\partial t}=\theta k_{2}\Psi C$$

(12) $$\Psi =1-\frac{S_{2}}{S_{max2}}$$

where *θ* is the volumetric water content; *C* is the solute concentration in the liquid phase (mmol·L^−1^); *ρ_b_* is the bulk density (g·cm^−3^); *v* is the Darcy velocity (cm·min^−1^); *S*_1_ and *S*_2_ are the sorbed concentrations on reversible sites (site 1) and irreversible/capacity-limited sites (site 2), respectively (mmol·g^−1^); *D* is the dispersion coefficient (cm^2^·min^−1^); *k*_1_ and *k*_1*d*_ are the first-order attachment and detachment rate constants for site 1 (min^−1^); *k*_2_ is the first-order attachment rate constant for site 2 (min^−1^); Ψ is a time-dependent Langmuir blocking factor for site 2; and *S*_*max*2_ is the maximum sorption capacity on site 2 (mmol·g^−1^).

## 3. Results and Discussion

### 3.1. Elemental Composition and Properties

The elemental compositions of the four natural manganese minerals are presented in Table 1. The results reveal significant differences in Fe contents and Mn/Fe ratios among the four minerals, although FM1 and FM2 showed the same measured Mn content. FM1 is characterized by a high Mn content (27.24%) and low Fe content (1.20%), resulting in a high Mn/Fe mass ratio of 22.7. FM2 also showed a Mn content (27.24%), as determined by acid digestion followed by ICP-OES, but had a substantially higher Fe content (6.53%), yielding a Mn/Fe ratio of 4.2. FM3 contains slightly lower Mn (22.08%) and 5.91% Fe (Mn/Fe = 3.7). FM4 exhibits the lowest Mn and Fe contents, at 11.81% and 0.72%, respectively (Mn/Fe = 16.4). Additionally, all minerals contain high proportions of Ca (53.06–79.53%) and Mg (2.03–10.24%), along with minor amounts of Al, Na, K, and trace levels of Cr and Zn. The Mn/Fe ratios and associated Ca/Mg contents vary considerably among the four minerals.

**Table 1:** Elemental Analysis of the Samples.

Sample	Mn	Fe	Ca	Al	Na	Mg	Cr	K	Zn
FM1	27.24	1.20	66.88	1.78	0.44	2.03	0.01	0.30	0.07
FM2	27.24	6.53	53.06	1.91	0.32	10.24	0.06	0.51	0.05
FM3	22.08	5.91	59.78	2.66	0.13	8.73	0.04	0.50	0.06
FM4	11.81	0.72	79.53	0.44	0.33	7.01	0.01	0.10	0.06

Only elements with a percentage content greater than 0.01% are shown in the table.

Table 2 shows that FM1 possesses a significantly larger specific surface area (22.98 m^2^·g^−1^) and pore volume (0.0666 cm^3^·g^−1^) compared to the other minerals, indicating a more developed pore structure and a greater abundance of potential adsorption sites. In contrast, the porosity values of the four minerals range only from 0.47 to 0.59, suggesting that the differences in overall pore space among the packed samples are relatively small. The pH at the point of zero charge (pH_PZC_) for the four minerals ranges from 2.40 to 3.46, indicating that their surfaces are negatively charged under neutral to weakly alkaline conditions. FM1 has the highest pH_PZC_ among the four minerals; therefore, at a given pH above pH_PZC_, the surface of FM1 is comparatively less negatively charged. This difference may influence the interfacial electrostatic environment, but it should not be regarded as the dominant control on As(V) uptake, which is more likely governed by specific surface complexation, especially inner-sphere complexation, on Fe/Mn-bearing sites [45].

**Table 2:** Pore Structure Parameters and pH_PZC_ of the 4 Samples.

Sample	Specific Surface Area (m^2^·g^−1^)	Pore Volume (cm^3^·g^−1^)	Pore Diameter (nm)	Porosity	pH_PZC_
FM1	22.98	0.0666	13.22	0.53	3.46
FM2	2.45	0.0113	21.84	0.51	2.54
FM3	4.14	0.0149	12.80	0.47	2.76
FM4	3.00	0.0094	10.14	0.59	2.40

### 3.2. Adsorption Characteristics

#### 3.2.1. Adsorption Isotherms

**Figure 2:**
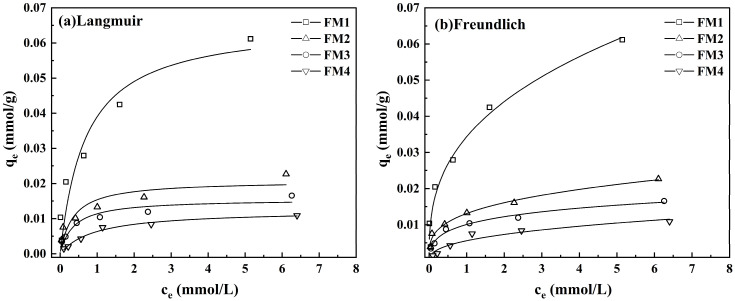
Isothermal adsorption experimental data and the fitting curves by (**a**) Langmuir isotherms, (**b**) Freundlich isotherms, respectively.

The adsorption isotherms for As(V) onto the four manganese minerals are shown in Figure 2. The equilibrium adsorption capacity (*q*_e_) of all minerals increased with the initial As(V) concentration. At a given equilibrium concentration (*C*_e_), the adsorption capacity followed the order: FM1 > FM2 > FM3 > FM4. The experimental data were fitted to the Freundlich and Langmuir models by nonlinear regression of the original *q*_e_-*C*_e_ data, with the fitted parameters presented in Table 3. As shown in Figure 2 and Table 3, the isotherms for FM1, FM2, and FM3 were better described by the Freundlich model (R^2^ = 0.957–0.981), suggesting that adsorption onto these minerals is primarily dominated by multi-layer chemical adsorption, this is consistent with the findings of Tian et al. [46]. In contrast, the isotherm for FM4 was better fitted by the Langmuir model (R^2^ = 0.980), indicative of monolayer adsorption, this may indicate that the number of available adsorption sites is relatively limited. The maximum adsorption capacities (*q*_m_) derived from the Langmuir model were 0.066, 0.021, 0.015, and 0.012 mmol·g^−1^ for FM1, FM2, FM3, and FM4, respectively. Correlating these values with the Mn and Fe contents and Mn/Fe ratios from Section 3.1, it is observed that the adsorption capacity was influenced not only by the Mn/Fe ratio, but also by the specific surface area, pore volume, and the total contents of Mn and Fe. For FM1–FM3, the adsorption capacity decreases as the Mn/Fe ratio decreases. In contrast, although FM4 had a relatively high Mn/Fe ratio, it showed the lowest uptake, likely because it also had the lowest Mn and Fe contents and the least developed pore structure.

Previous studies have shown that Fe–Mn adsorbents with more developed pore structures and higher specific surface areas generally exhibit markedly higher As(V) uptake. For example, mesoporous Fe–Mn oxides with optimized composition have been reported to show both large specific surface areas and high Langmuir capacities [47], while iron–manganese oxide chitosan gel microspheres also exhibited substantial As(V) uptake despite their smaller surface areas [48]. Although these synthetic materials are not directly comparable with the present natural minerals, the same general trend supports the superior performance of FM1, which had the highest specific surface area and pore volume among the four samples.

**Table 3:** Langmuir and Freundlich Model Parameters for As(V) Adsorption by the Manganese Ores.

Minerals	Langmuir			Freundlich		
	*q*_m_ mmol·g^−1^	*b*	R^2^	*K*_f_ (mmol·g^−1^)(L·mmol^−1^)^1/*n*^	*n*	R^2^
FM1	0.066	1.446	0.890	0.034	2.78	0.957
FM2	0.021	2.839	0.792	0.013	3.41	0.981
FM3	0.015	2.765	0.891	0.010	3.44	0.973
FM4	0.012	1.056	0.980	0.006	2.55	0.913

#### 3.2.2. Adsorption Kinetics

**Figure 3:**
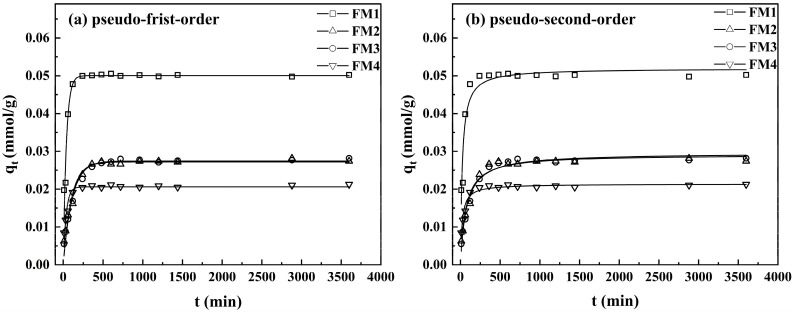
Kinetic adsorption experimental data and the fitting curves by (**a**) Pseudo-first-order kinetic, (**b**) Pseudo-second-order kinetic, respectively.

The adsorption kinetics of As(V) onto the four minerals and the corresponding model fits are presented in Figure 3 and Table 4. The adsorbed amount of As(V) increased rapidly with contact time before gradually approaching equilibrium. The adsorption process can be divided into three stages: an initial rapid phase (0–100 min), dominated by surface adsorption onto abundant active sites, resulting in the removal of most of the As(V) from solution; a subsequent slower phase (100–600 min), where adsorption transitions from surface binding to intra-particle pore diffusion as the solution concentration decreases; and a final equilibrium phase (after 600 min), where available sites become saturated. At equilibrium, FM1 exhibited the highest adsorption capacity (0.051 mmol·g^−1^), significantly greater than those of FM2 (0.028 mmol·g^−1^), FM3 (0.027 mmol·g^−1^), and FM4 (0.021 mmol·g^−1^). FM1 had the largest specific surface area (22.98 m^2^·g^−1^) and pore volume (0.0666 cm^3^·g^−1^), compared with 2.45, 4.14, and 3.00 m^2^·g^−1^ and 0.0113, 0.0149, and 0.0094 cm^3^·g^−1^ for FM2, FM3, and FM4, respectively. These direct comparisons indicate that the superior adsorption performance of FM1 was closely associated with its much larger accessible surface area and more developed pore structure.

The lower adsorption rates of FM2 and FM3 relative to FM1 can be attributed mainly to their much smaller specific surface areas and pore volumes, which limited the number and accessibility of active sites. In addition, their lower pH_PZC_ values imply a more negatively charged surface at the experimental pH, leading to stronger electrostatic repulsion toward arsenate anions. Although FM4 showed a relatively high apparent initial rate, this likely reflected the rapid occupation of a limited number of readily accessible surface sites rather than a higher overall adsorption capacity. The rapid initial uptake of As(V) followed by a slower approach to equilibrium observed in this study is consistent with adsorption on heterogeneous mineral surfaces, where external surface binding dominates the early stage and diffusion into internal domains becomes more important later [49,50].

The kinetic data were fitted to pseudo-first-order and pseudo-second-order models, with the derived parameters and coefficients of determination (R^2^) listed in Table 4. For FM1, both models yielded high R^2^ values (0.933 and 0.946), but the slightly better fit to the pseudo-second-order model suggests that the adsorption process involves not only mass transfer but also significant chemisorption [51]. For FM2, FM3, and FM4, the pseudo-second-order model provided superior fits (R^2^ = 0.979, 0.967, and 0.959, respectively) compared to the pseudo-first-order model, further confirming that multi-layer chemisorption is the dominant mechanism for these minerals. The pseudo-first-order rate constant (K_1_) for FM1 was 0.026 min^−1^, reflecting its rapid adsorption. Although FM4 exhibited the fastest initial rate (K_1_ = 0.027 min^−1^), the much smaller specific surface area (3.00 m^2^·g^−1^) and pore volume (0.0094 cm^3^·g^−1^) limited its overall uptake, resulting in the lowest equilibrium adsorption capacity among the four minerals.

**Table 4:** Fitting parameters of the pseudo-first-order and pseudo-second-order kinetic models.

Minerals	Pseudo-First-Order Kinetic			Pseudo-Second-Order Kinetic		
	*q*_e1_ mmol·g^−1^	*K*_1_ min^−1^	R^2^	*q*_e2_ mmol·g^−1^	*K*_2_ g·mmol·min^−1^	R^2^
FM1	0.051	0.026	0.933	0.052	0.861	0.946
FM2	0.028	0.009	0.973	0.030	0.462	0.979
FM3	0.027	0.010	0.960	0.029	0.521	0.967
FM4	0.021	0.027	0.902	0.021	2.270	0.959

### 3.3. Factors Influencing Adsorption

#### 3.3.1. Effect of Initial pH

**Figure 4:**
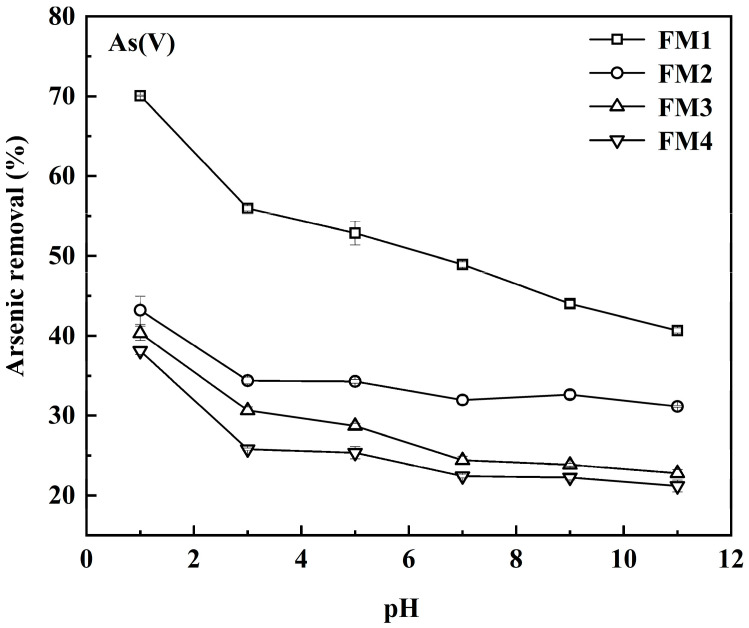
The adsorption behavior of As(V) under different pH conditions.

The initial solution pH significantly influences As(V) adsorption by controlling arsenic speciation and the surface charge of the minerals [52,53]. Arsenic acid dissociates with pK_1_ = 2.22, pK_2_ = 6.96, and pK_3_ = 11.50. Thus, As(V) exists predominantly as H_2_AsO_4_^−^ at pH > 2.22, as HAsO_4_^2−^ at pH > 6.96, and as AsO_4_^3−^ at pH > 11.50. The pH_PZC_ of the four minerals ranges from 2.40 to 3.46 (Table 2); their surfaces are positively charged at pH < pH_PZC_, favoring As(V) anion adsorption, and negatively charged at pH > pH_PZC_, which is unfavorable. As shown in Figure 4, the removal efficiency of As(V) by all four minerals generally decreased as the initial pH increased from 1 to 11. Although pH 1 and 11 represent boundary conditions rather than environmentally typical conditions, they were retained to reveal the overall pH sensitivity of the four minerals. Within the more environmentally relevant near-neutral range, the same decreasing trend remained evident. Previous studies have indicated that while arsenate adsorption may be more favorable under low pH conditions, the stability of iron (hydro)oxides decreases under acidic conditions. The removal rate of approximately 70% observed at initial pH 1 in Figure 4 should be regarded as a short-term apparent removal rate under batch test conditions, rather than evidence of long-term stable immobilization [54]. At initial pH 3, removal efficiencies decreased markedly for all minerals, though FM1 retained relatively good performance. As initial pH increased further to 11, removal efficiencies continued to decline. FM1 showed the most pronounced decrease (approximately 15%), while the removal efficiencies of the other three minerals decreased more slowly, stabilizing between 21% and 32%. Wu et al. reported that As(V) adsorption by muscovite-supported iron-manganese oxides decreased substantially as pH increased from 3 to 9, and attributed this behavior to lower surface protonation and stronger competition from OH^−^ for adsorption sites at higher pH [55]. The decline at higher pH is attributed to increased negative charge on the mineral surfaces and competition for adsorption sites from OH^−^ ions. Throughout the entire pH range, FM1 exhibited the highest removal efficiency. This superior performance can be linked to its larger specific surface area, greater pore volume (more developed mesoporous structure), and higher pH_PZC_ (Table 2). These structural and surface charge characteristics collectively reduce electrostatic repulsion and enhance surface complexation, facilitating greater As(V) immobilization [56,57].

#### 3.3.2. Desorption and Regeneration

**Figure 5:**
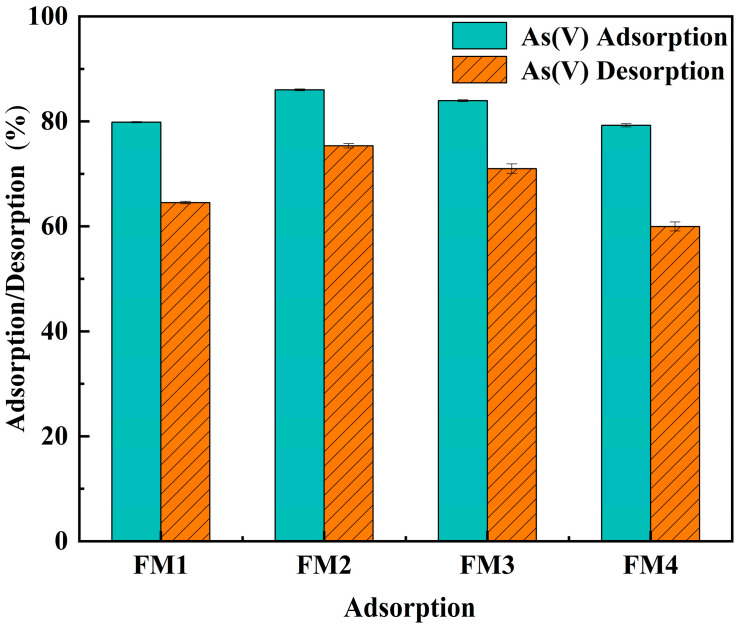
Adsorption and desorption behavior of As(V) on natural manganese mineral adsorbents.

According to Nikić et al. and Vujić et al., 0.1 M NaOH is the most suitable regenerant, as it provides optimal desorption performance while minimizing adsorbent degradation [58]. To evaluate the reversibility and binding strength of adsorbed As(V), desorption experiments were conducted using 0.1 mol·L^−1^ NaOH, with results shown in Figure 5. The initial As(V) removal efficiencies for FM1, FM2, FM3, and FM4 were 79.9%, 85.9%, 83.8%, and 79.5%, respectively, indicating good adsorption performance across all four minerals. It should be noted that the initial removal efficiency under a fixed experimental condition does not necessarily follow the same order as the maximum adsorption capacity (*q*_m_). The *q*_m_ value obtained from the Langmuir model represents the maximum adsorption capacity under near-saturation conditions, whereas the initial removal efficiency is more strongly influenced by the proportion of readily accessible sites and the short-term adsorption behavior at the tested initial concentration. Thus, although FM1 showed the highest *q*_m_, FM2 and FM3 exhibited slightly higher initial removal efficiencies under the specific desorption-test condition. The subsequent desorption efficiency, which directly reflects the binding strength between As(V) and the surface active sites, varied significantly among the minerals. Desorption efficiencies were 64.7% for FM1, 75.0% for FM2, 71.6% for FM3, and 59.4% for FM4. Treatment with NaOH facilitates desorption through two main mechanisms: (1) direct displacement of adsorbed As(V) anions (e.g., HAsO_4_^2−^) by OH^−^ via competitive binding, and (2) increasing the negative surface charge, which generates strong electrostatic repulsion against As(V) anions. Notably, FM2, despite having a high initial adsorption capacity, also exhibited a high desorption rate, suggesting relatively weak binding dominated by physical adsorption. Conversely, FM4 showed stronger binding (lower desorption) but had a limited overall adsorption capacity. Considering both adsorption performance and desorption stability, FM1 is inferred to form relatively stable inner-sphere surface complexes with As(V) under dynamic conditions. These complexes likely involve direct M–O–As (M = Fe or Mn) linkages formed through ligand exchange with surface hydroxyl groups, which can enhance the stability of adsorbed As(V) and favor more sustained arsenic immobilization [59].

### 3.4. Effluent Chemistry Dynamics

#### 3.4.1. Arsenic Breakthrough

**Figure 6:**
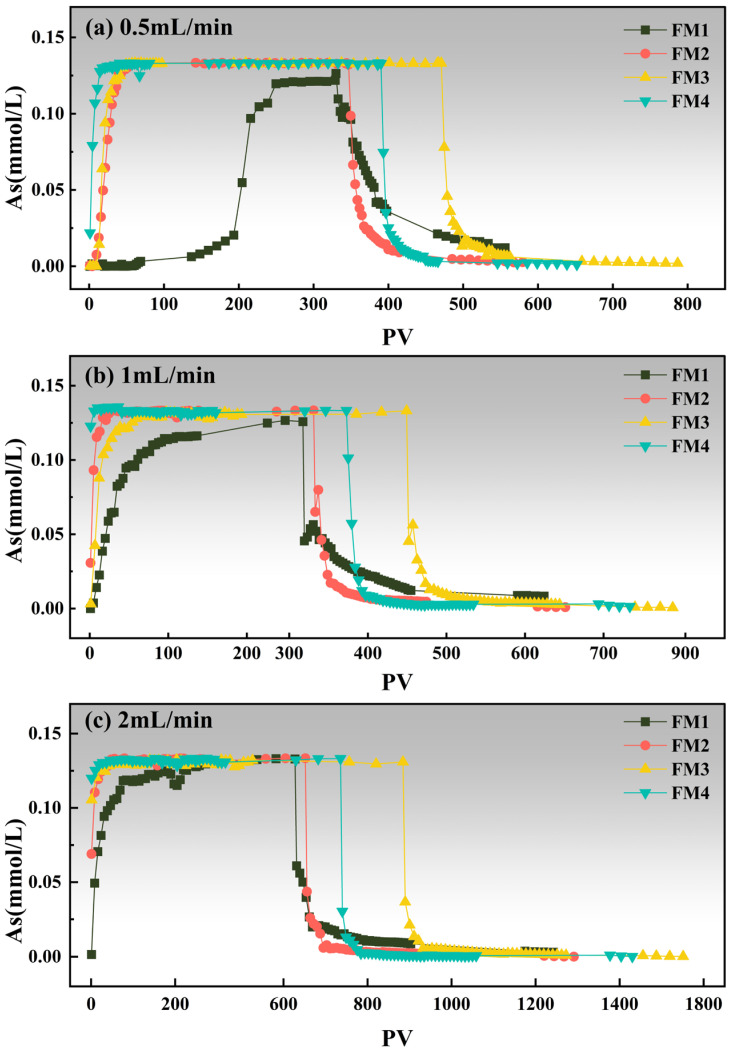
Changes in As concentrations in the effluent solution from four mineral columns at different flow rates, expressed as a function of pore volume (PV).

Effluent As concentrations for the four mineral columns at different flow rates are shown in Figure 6 as a function of pore volume (PV). At 0.5 mL·min^−1^ (Figure 6a), FM4 showed the earliest breakthrough, with effluent As approaching the influent level (0.13 mmol·L^−1^) at about PV 14.4, followed by FM3 at PV 33.3 and FM2 at PV 39.3. In contrast, FM1 exhibited substantially delayed breakthrough, reaching approximately 0.12 mmol·L^−1^ only at PV 261.1, indicating much stronger retardation under low-flow conditions. After the influent was switched, the desorption branches of FM2–FM4 declined rapidly, whereas FM1 maintained the longest and most pronounced tail. The transition from plateau to decline occurred at about PV 329.9 for FM1, 346.8 for FM2, 474.7 for FM3, and 390.1 for FM4, again highlighting the different pore-volume-normalized transport behaviors among the minerals. This pattern is consistent with fixed-bed studies showing that lower flow rate and longer effective bed contact time can significantly delay arsenic breakthrough in Fe–Mn-based adsorbents [60].

At 1.0 mL·min^−1^ (Figure 6b), FM4 again showed the most rapid breakthrough, with effluent As approaching 0.13 mmol·L^−1^ at about PV 1.1, followed by FM2 at PV 16.7 and FM3 at PV 38.7. FM1 remained the most retarded column, reaching approximately 0.12 mmol·L^−1^ only at PV 272.1, which is close to its value at 0.5 mL·min^−1^. This behavior does not contradict the rapid uptake of FM1 observed in the batch kinetic experiment, because the batch system mainly reflects fast adsorption on readily accessible sites under well-mixed conditions, whereas column retardation under flow also depends on residence time, intra-particle diffusion, and the effective utilization of slower retention sites. After influent switching, the decline branches began at different PV values, approximately 319.2 for FM1, 333.0 for FM2, 452.0 for FM3, and 375.0 for FM4. Among the four minerals, FM1 still retained the most evident desorption tail, whereas FM2–FM4 declined much more rapidly. These results indicate that, even after PV normalization, FM1 sustained stronger retardation and slower release than the other three minerals. Similar flow-rate effects on arsenic fixed-bed breakthrough behavior have also been reported in continuous column studies [61].

At 2.0 mL·min^−1^ (Figure 6c), all columns showed comparatively rapid breakthrough, but the breakthrough PVs still differed among minerals. FM4 and FM3 approached the influent As concentration at about PV 10.0 and PV 12.0, respectively, followed by FM2 at PV 24.5. FM1 remained the most retarded column, but its breakthrough PV decreased markedly to PV 136.8, much lower than at 0.5 and 1.0 mL·min^−1^. After the switch in influent, the decline branches started at around PV 631.1 for FM1, 655.9 for FM2, 889.6 for FM3, and 740.0 for FM4. Although all curves then declined rapidly, FM1 still showed a visible tail, whereas FM2–FM4 approached near-baseline concentrations more quickly. This indicates that the highest flow rate compressed the mass-transfer zone and reduced the effective utilization of slower retention sites.

Overall, when expressed on a PV basis, the breakthrough behavior did not decrease monotonically with increasing flow rate for all minerals. FM1 and FM3 showed slightly larger breakthrough PV values at 1.0 than at 0.5 mL·min^−1^, whereas FM2 and FM4 displayed non-monotonic responses. This indicates that breakthrough PV is controlled not only by retention strength, but also by front shape, hydrodynamic dispersion, and the effective use of fast and slow retention sites. Nevertheless, the overall performance ranking remained clear: FM1 consistently exhibited the strongest retardation and the most pronounced desorption tail, whereas FM2–FM4 approached the influent concentration much more rapidly. Previous fixed-bed and pilot-scale studies likewise show that arsenic breakthrough is jointly controlled by flow rate, empty bed contact time (EBCT), and matrix-/transport-related effects, rather than by a single operational parameter alone [60,62].

#### 3.4.2. Iron and Manganese Release

**Figure 7:**
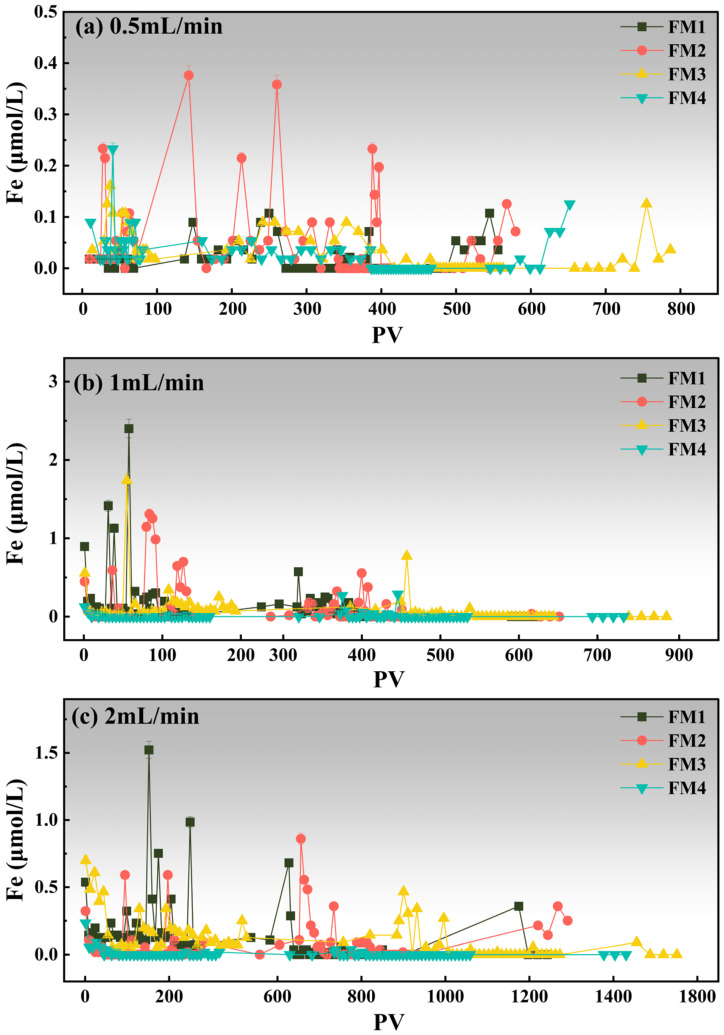
Changes in Fe concentrations in the effluent solution from four mineral columns at different flow rates, expressed as a function of pore volume (PV).

**Figure 8:**
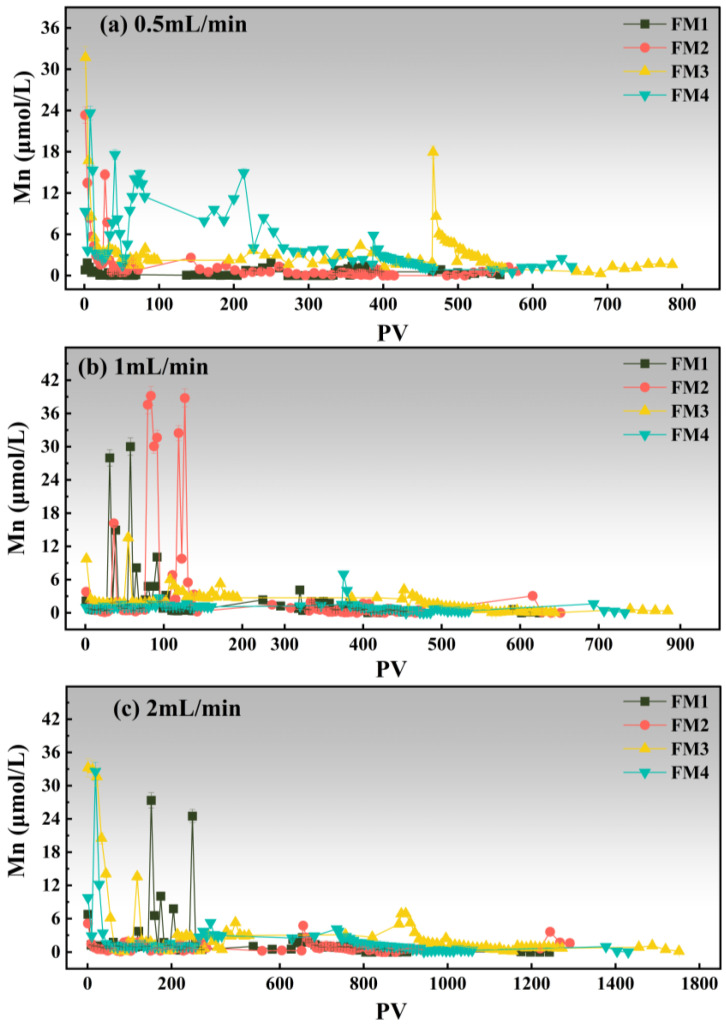
Changes in Mn concentrations in the effluent solution from four mineral columns at different flow rates, expressed as a function of pore volume (PV).

Following the As breakthrough analysis, the release behaviors of Fe and Mn were further examined under the same column conditions. The Fe release curves are shown in Figure 7, and the Mn release curves are shown in Figure 8.

Fe release remained generally low across all columns and was mainly expressed as brief, transient peaks rather than sustained dissolution (Figure 7). At 0.5 mL·min^−1^ (Figure 7a), FM2 showed the most prominent Fe fluctuations, with two peaks reaching about 0.38 μmol·L^−1^ at PV 124.3 and 0.36 μmol·L^−1^ at PV 245.6, whereas FM1, FM3, and FM4 only exhibited minor peaks, with maxima of about 0.11 μmol·L^−1^ at PV 245.6, 0.16 μmol·L^−1^ at PV 13.3, and 0.23 μmol·L^−1^ at PV 11.0, respectively. At 1.0 mL·min^−1^ (Figure 7b), Fe release increased for FM1–FM3, and FM1 showed the highest peak (2.40 μmol·L^−1^ at PV 57.4), followed by FM3 (1.74 μmol·L^−1^ at PV 54.7) and FM2 (1.31 μmol·L^−1^ at PV 83.5); FM4 remained consistently low. At 2.0 mL·min^−1^ (Figure 7c), FM1 again displayed the largest transient peak (1.52 μmol·L^−1^ at PV 151.9), whereas FM2 and FM3 reached maxima of 0.86 μmol·L^−1^ at PV 655.9 and 0.70 μmol·L^−1^ at PV 1.3, respectively, and FM4 still remained low. Overall, Fe release was limited and highly transient, suggesting that Fe-bearing phases in these minerals were comparatively stable under column operation, and that most released Fe likely originated from weakly bound or exchangeable surface-associated Fe rather than continuous bulk dissolution. This interpretation is broadly consistent with previous studies on Fe–Mn-based arsenic adsorbents, which reported that Fe release is generally weaker and more controllable than Mn release under column conditions [63,64].

Mn release was much more pronounced than Fe release and showed stronger dependence on both mineral type and flow rate (Figure 8). At 0.5 mL·min^−1^ (Figure 8a), FM1 maintained comparatively low Mn concentrations for most of the run, although several moderate peaks appeared later. In contrast, FM2 and FM3 showed strong initial release, with maxima of about 23.34 μmol·L^−1^ at PV 1.0 and 31.71 μmol·L^−1^ at PV 1.3, respectively. FM4 exhibited the most sustained release pattern, with Mn concentrations remaining elevated over a broad PV range and reaching values above 18.20 μmol·L^−1^. At 1.0 mL·min^−1^ (Figure 8b), Mn release intensified further for FM1 and FM2. FM1 displayed two major peaks of 27.98 μmol·L^−1^ at PV 31.1 and 30.01 μmol·L^−1^ at PV 57.4, whereas FM2 showed the most vigorous release overall, with multiple peaks above 27.31 μmol·L^−1^, including maxima of about 37.58 μmol·L^−1^ at PV 79.6 and 39.18 μmol·L^−1^ at PV 83.5. FM3 showed moderate but still evident release, while FM4 remained relatively low at this flow rate. At 2.0 mL·min^−1^ (Figure 8c), FM1 again exhibited two high peaks (27.345 μmol·L^−1^ at PV 151.9 and 24.506 μmol·L^−1^ at PV 250.0). FM3 showed the strongest initial pulse (33.22 μmol·L^−1^ at PV 1.3) followed by a rapid decline and later secondary release around PV 889.6. FM4 also exhibited a pronounced early peak (32.59 μmol·L^−1^ at PV 18.8) but remained low thereafter. Overall, Mn release was consistently more intense and more variable than Fe release, indicating greater lability of Mn-bearing phases. Previous studies on Mn-oxide-containing and Fe–Mn-based adsorbents have likewise shown that Mn release is a key stability issue during arsenic removal in filter columns, because Mn phases are more reactive and more mobile than Fe phases under dynamic flow conditions [64,65]. The four minerals showed clear differences in As breakthrough as well as Fe and Mn release behavior under flow conditions. Fe release remained generally low, whereas Mn release was more pronounced and more sensitive to mineral type and flow rate. These results indicate differences in transport-retention performance and structural stability among the minerals, but they do not by themselves demonstrate a direct interaction among As, Fe, and Mn.

### 3.5. Breakthrough Curve Analysis and Modeling

#### 3.5.1. Bromide Breakthrough Curves

In transport studies, conservative tracers are commonly used to constrain hydrodynamic parameters prior to modeling reactive solute transport. Because Br^−^ interacts only weakly with Mn/Fe oxides, it was used here as a conservative tracer to characterize hydrodynamic transport rather than As(V) adsorption behavior [41]. The Br^−^ breakthrough curves were analyzed using the one-dimensional Advection-Dispersion Equation (ADE), from which the hydrodynamic dispersion coefficient (D) was determined [42]. These parameters were then used to constrain the subsequent modeling of reactive As(V) transport.

**Figure 9:**
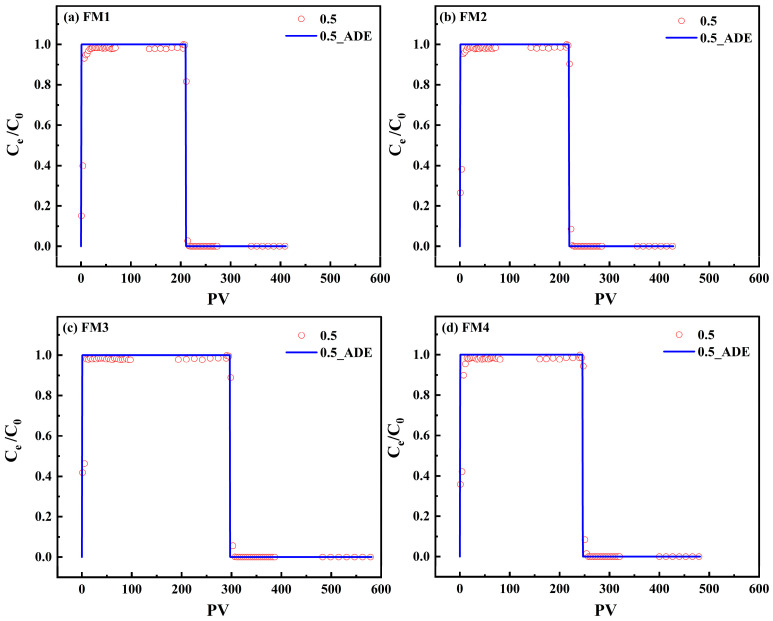
Experimental and ADE model-simulated breakthrough and elution curves of the Br^−^ tracer for different adsorbents.

At a flow rate of 0.5 mL·min^−1^, the Br^−^ BTCs obtained from columns packed with FM1, FM2, FM3, and FM4 were highly similar when plotted against PV (Figure 9a–d). All curves exhibited rapid breakthrough, a near-unity plateau, and a sharp decline during elution, indicating typical conservative transport behavior with minimal retention in the solid phase. ADE fitting reproduced these BTCs reasonably well, with R^2^ values ranging from 0.8910 to 0.9108 and low RMSE values of 0.0116–0.0202 (Table 5). The fitted D values were 0.12, 0.13, 0.11, and 0.12 cm^2^·min^−1^ for FM1, FM2, FM3, and FM4, respectively. Because the packed columns had similar porosities (Table 2) and the Br^−^ BTCs were nearly overlapping in PV coordinates, the variation in D among minerals was considered secondary relative to the effect of flow rate. These results indicate that, at 0.5 mL·min^−1^, mineral type had little influence on conservative tracer transport under the present packing and hydraulic conditions.

**Figure 10:**
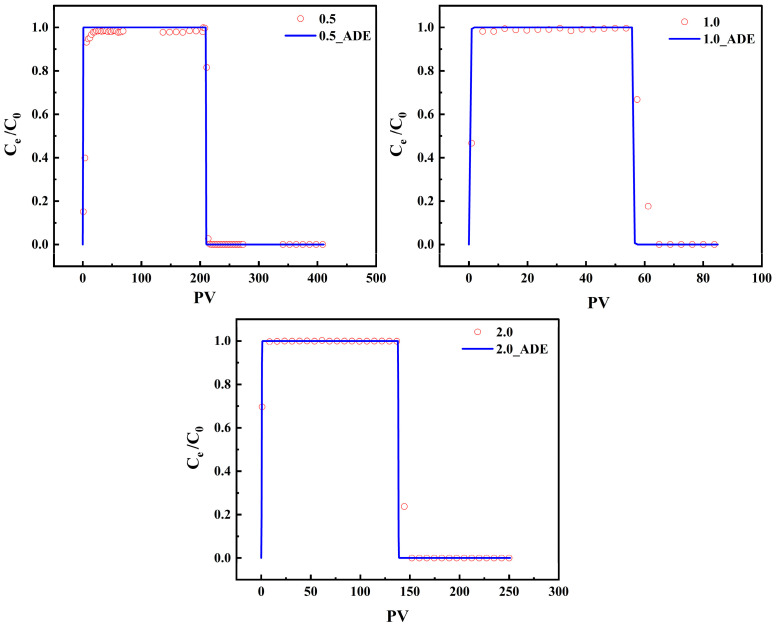
Adsorption-desorption of Br^−^ tracer by FM1 at three flow rates with ADE model fitting results.

Tracer experiments with Br^−^ were subsequently conducted in the FM1 column at flow rates of 0.5, 1.0, and 2.0 mL·min^−1^, and the BTCs were fitted using the ADE model (Figure 10, Table 5). In PV coordinates, all three BTCs showed rapid breakthrough and elution, confirming the conservative nature of Br^−^ transport and the suitability of PV normalization for comparing flow-dependent behavior.

As the flow rate increased from 0.5 to 1.0 to 2.0 mL·min^−1^, the fitted D value increased correspondingly from 0.12 to 0.21 to 0.43 cm^2^·min^−1^. This positive relationship is physically reasonable and reflects enhanced mechanical dispersion at higher pore-water velocities [66]. Although the R^2^ values decreased slightly with increasing flow rate (0.8913, 0.8800, and 0.8552), the RMSE values remained low (0.0202, 0.0090, and 0.0080), indicating that the ADE adequately captured the main features of Br^−^ transport across the tested hydraulic conditions. These hydrodynamic parameters provide the basis for constraining the subsequent modeling of reactive As(V) transport.

**Table 5:** ADE model-fitted parameters for Br^−^ tracer transport at different flow rates.

Minerals-*v*	*D* (cm^2^·min^−1^)	R^2^	RMSE
FM1-0.5	0.12	0.8913	0.0202
FM2-0.5	0.13	0.8910	0.0151
FM3-0.5	0.11	0.9108	0.0116
FM4-0.5	0.12	0.8963	0.0134
FM1-1	0.21	0.8800	0.0090
FM1-2	0.43	0.8552	0.0080

*D* denotes the hydrodynamic dispersion coefficient constrained from Br^−^ tracer BTCs and fixed in the subsequent As(V) transport simulations at the corresponding flow rate.

#### 3.5.2. Arsenic Breakthrough Curves

**Figure 11:**
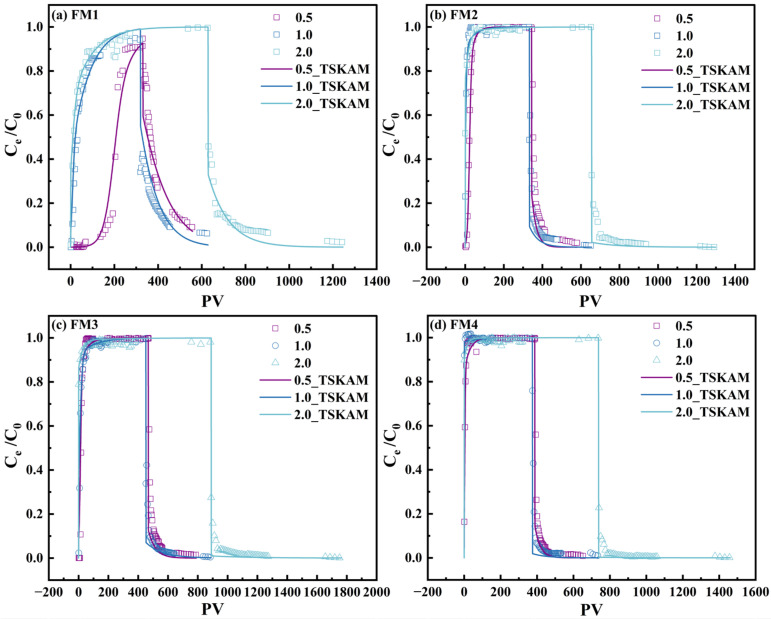
Breakthrough curves of As(V) in four natural manganese minerals at different flow rates and their fitting with the TSKAM.

To elucidate the effect of flow rate on As(V) transport, column experiments were conducted at 0.5, 1.0, and 2.0 mL·min^−1^ for each mineral. The resulting BTCs, expressed as *C*_e_/*C*_0_ versus PV, and their TSKAM fits are shown in Figure 11, with the fitted parameters summarized in Table 6. Following the concept of two-site nonequilibrium transport models [67], the TSKAM was used here to represent fast and slow retention processes of As(V) in the mineral columns. It should also be noted that D was not independently optimized during As(V) fitting; instead, it was fixed at the value constrained from the Br^−^ tracer BTCs at the corresponding flow rate.

FM1 exhibited the strongest retardation among the four minerals (Figure 11a). In PV coordinates, breakthrough (*C*_e_/*C*_0_ ≥ 0.9) occurred at PV 261.1, 272.1, and 136.8 at flow rates of 0.5, 1.0, and 2.0 mL·min^−1^, respectively. Thus, the breakthrough PV did not decrease monotonically with increasing flow rate. The slight increase from 0.5 to 1.0 mL·min^−1^ suggests that, after PV normalization, enhanced external mass transfer and front broadening may partly offset the reduced residence time, whereas at 2.0 mL·min^−1^ the residence-time limitation became dominant. Such behavior is consistent with recent discussions showing that breakthrough-curve shape and the selected breakthrough criterion can be influenced by both hydrodynamic dispersion and nonequilibrium retention, rather than by sorption strength alone. The TSKAM still fitted the FM1 BTCs well (R^2^ = 0.9486–0.9821, RMSE < 0.03), and the marked decrease in *S*_*max*2_ from 1.20 to 0.05 mg·g^−1^ indicates a strong reduction in slow, capacity-limited retention at higher flow rate [26,68,69].

FM2, FM3, and FM4 did not show a simple monotonic decrease in breakthrough PV with increasing flow rate (Figure 11b–d). For FM2, breakthrough occurred at PV 39.3, 16.7, and 24.5 at 0.5, 1.0, and 2.0 mL·min^−1^, respectively, indicating an initial decrease followed by a partial rebound. For FM3, the corresponding breakthrough PV values were 33.3, 38.7, and 12.0, i.e., a slight increase from 0.5 to 1.0 mL·min^−1^ followed by a marked decrease at 2.0 mL·min^−1^. For FM4, the breakthrough PV values were 14.4, 1.1, and 10.0, showing a sharp drop at 1.0 mL·min^−1^ and then a moderate increase at 2.0 mL·min^−1^. These contrasting patterns indicate that the PV at *C*_e_/*C*_0_ = 0.9 is controlled not only by retardation strength, but also by front shape, hydrodynamic dispersion, and the relative contribution of fast and slow retention sites. Similar complexity in arsenic breakthrough behavior under column conditions has been reported in recent experimental-modeling studies, especially where transport reflects coupled effects of reactive media, pH, and sediment heterogeneity [70,71]. TSKAM still fitted these BTCs well, with R^2^ values of 0.9330–0.9887 for FM2, 0.9087–0.9947 for FM3, and 0.9432–0.9967 for FM4.

Overall, the results indicate that the breakthrough PV at *C*_e_/*C*_0_ = 0.9 should not be interpreted as a monotonic function of flow rate for all minerals. Instead, it reflects the combined effects of residence time, hydrodynamic dispersion, front steepness, and nonequilibrium site utilization. By contrast, the fitted *S*_*max*2_ values show a much clearer trend, decreasing from 1.20 to 0.05 for FM1, 0.15 to 0.03 for FM2, 0.07 to 0.02 for FM3, and 0.02 to 0.01 for FM4 as the flow rate increased from 0.5 to 2.0 mL·min^−1^. This consistently supports the conclusion that higher flow rate suppresses the contribution of the slow, capacity-limited site, even though the breakthrough PV itself may shift non-monotonically. FM1 still exhibited the highest *S*_*max*2_ and the largest breakthrough PV values overall, consistent with its larger specific surface area and pore volume, while its higher pH_PZC_ implies a less negative surface under circumneutral conditions, which is more favorable for As(V) retention. Recent work on natural Mn–Fe binary materials and Fe–Mn oxide systems likewise indicates that arsenic retention is jointly controlled by surface properties, pH, and Mn/Fe-related reactivity.

Although the batch and column results showed generally similar trends, the fitted parameters from the two systems are not directly comparable because they describe different processes under different hydrodynamic conditions. The batch pseudo-first-order and pseudo-second-order parameters characterize overall uptake under well-mixed conditions, whereas the TSKAM parameters describe transport-coupled retention under flow conditions. Nevertheless, the overall performance ranking was consistent: FM1 showed the highest equilibrium uptake in the batch experiments and the strongest retardation in the column experiments, whereas FM4, despite a relatively rapid apparent initial uptake in batch, exhibited the lowest equilibrium capacity and the weakest column retention. FM2 and FM3 showed intermediate behavior in both systems.

**Table 6:** TSKAM fitted parameters for adsorption–desorption of As(V) by four natural manganese minerals at different flow rates.

Minerals-*v*	*D* (cm^2^·min^−1^)	*k*_1_ (min^−1^)	*k*_1d_ (min^−1^)	*k*_2_ (min^−1^)	*S*_max2_ (mg·g^−1^)	R^2^	RMSE
FM1-0.5	0.12	0.210	0.0015	1.7	1.20	0.9486	0.0241
FM2-0.5	0.13	0.055	0.0045	0.9	0.15	0.9330	0.0599
FM3-0.5	0.11	0.050	0.0030	0.9	0.07	0.9087	0.0263
FM4-0.5	0.12	0.040	0.0040	0.4	0.02	0.9432	0.0384
FM1-1	0.21	0.310	0.0035	0.7	0.05	0.9676	0.0290
FM2-1	0.23	0.040	0.0045	0.3	0.02	0.9693	0.0360
FM3-1	0.21	0.055	0.0025	0.7	0.05	0.9745	0.0094
FM4-1	0.20	0.010	0.0030	0.0	0.0	0.9630	0.0303
FM1-2	0.43	0.300	0.0050	0.5	0.05	0.9821	0.0004
FM2-2	0.41	0.018	0.0030	0.2	0.03	0.9887	0.0149
FM3-2	0.40	0.015	0.0020	0.2	0.02	0.9947	0.0048
FM4-2	0.41	0.003	0.0020	0.1	0.01	0.9967	0.0046

### 3.6. Characterization Results

#### 3.6.1. XRD

**Figure 12:**
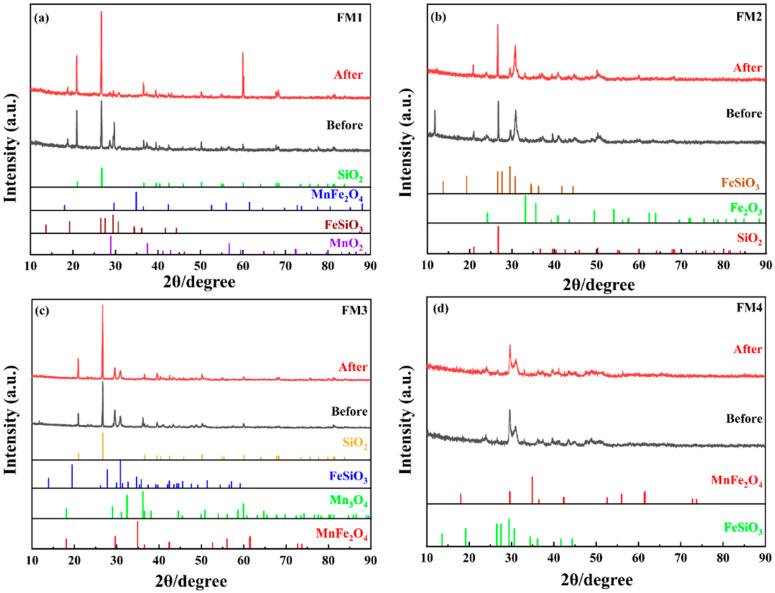
XRD patterns of different adsorbents before and after As(V) adsorption. (**a**) FM1; (**b**) FM2; (**c**) FM3; (**d**) FM4.

To gain a deeper understanding of the mechanisms by which natural manganese minerals with different Mn/Fe ratios adsorb As(V), batch isotherm and kinetic data alone are insufficient. Therefore, this study employed a combination of characterization techniques, including Fourier-transform infrared spectroscopy (FTIR), X-ray diffraction (XRD), X-ray photoelectron spectroscopy (XPS), and scanning electron microscopy with energy-dispersive X-ray spectroscopy (SEM-EDS). These analyses were performed on mineral samples before and after the column experiments to investigate changes in functional groups, crystal structure, elemental valence states, and surface morphology, thereby provide further information on the adsorption process. Figure 12 shows the XRD patterns of the four minerals before and after As(V) adsorption. Before the reaction, FM1 was primarily composed of SiO_2_, MnFe_2_O_4_, FeSiO_3_, and MnO_2_. After As(V) adsorption, the main mineral phases remained broadly stable, but the diffraction peaks at 2*θ* = 28.7° and 29.7°, corresponding to FeSiO_3_ and MnO_2_, respectively, showed a slight decrease in intensity (Figure 12a). This suggests that Fe/Mn-bearing phases may have participated in the adsorption process at their surfaces, although the current XRD data alone do not allow a more specific distinction between surface adsorption, local surface alteration, or attenuation of diffraction signals by adsorbed surface species. Pristine FM2 consisted mainly of SiO_2_, FeSiO_3_, and Fe_2_O_3_. After the reaction, the intensity of the Fe_2_O_3_ peaks decreased (Figure 12b), indicating possible surface involvement of Fe-bearing phases during adsorption. Pristine FM3 was composed of SiO_2_, MnFe_2_O_4_, FeSiO_3_, and Mn_3_O_4_. Following As(V) adsorption, the intensities of the peaks at 2*θ* = 29.5° and 36.2°, corresponding to Mn_3_O_4_ and MnFe_2_O_4_, respectively, were slightly reduced (Figure 12c). Pristine FM4 was mainly composed of FeSiO_3_ and MnFe_2_O_4_. After the reaction, a slight decrease in the intensity of the MnFe_2_O_4_ diffraction peaks was observed (Figure 12d).

Overall, the XRD results indicate that the major crystalline phases of the four minerals remained identifiable before and after adsorption, suggesting that no pronounced bulk phase transformation occurred during the experiments. The slight weakening of some Fe/Mn-related diffraction peaks implies that these phases may have been involved in the adsorption process, but the present XRD evidence by itself is not sufficient to establish a specific reaction pathway. Because no quartz-only blank experiment or dissolved-silica measurement was conducted in this study, the possible influence of quartz dissolution and silicic-acid competition cannot be independently evaluated from the current dataset and is therefore not further inferred here. FM1 still clearly displayed the characteristic diffraction peaks of MnFe_2_O_4_ before and after reaction, indicating the persistence of this Mn-Fe oxide phase during adsorption. XRD results mainly suggest that Fe/Mn-bearing crystalline phases remained present and may have contributed to As(V) uptake, whereas the detailed adsorption mechanism is further discussed in the following sections.

#### 3.6.2. FTIR

**Figure 13:**
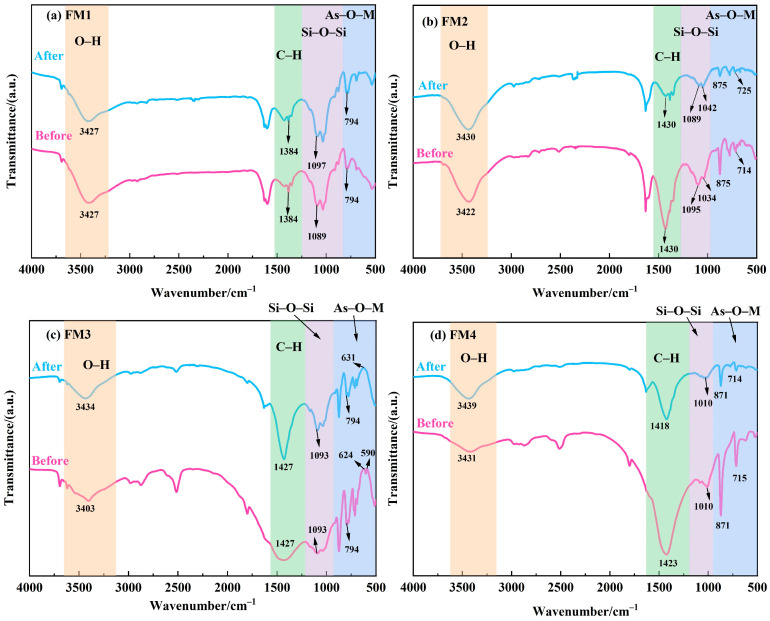
FTIR spectra of different adsorbents before and after As(V) adsorption.

The FTIR spectra of the four minerals before and after As(V) adsorption are presented in Figure 13. The spectrum of pristine FM1 (Figure 13a) shows four main absorption bands: a broad band near 3427 cm^−1^, attributed to O–H stretching vibrations; a band at 1384 cm^−1^, corresponding to C–H vibrations from aliphatic or organic matter; a strong band near 1089 cm^−1^, assigned to Si–O–Si asymmetric stretching; and a band at 794 cm^−1^, characteristic of As–O–M (M = Fe, Mn) stretching. After As(V) adsorption, the intensity of the 1384 cm^−1^ band decreased, suggesting the involvement of organic matter. The Si–O–Si band shifted from 1089 cm^−1^ to 1097 cm^−1^, and the intensity of the As–O–M band at 794 cm^−1^ increased, indicating complexation of As with Fe/Mn–O groups, consistent with the XRD findings. The spectra for FM2, FM3, and FM4 (Figure 13b–d) exhibited similar features. For FM2, after reaction, the O–H band shifted from 3422 cm^−1^ to 3430 cm^−1^, the C–H band at 1430 cm^−1^ decreased significantly, and the As–O–M band shifted from 714 cm^−1^ to 725 cm^−1^ with increased intensity at 875 cm^−1^. For FM3, the O–H band shifted from 3403 cm^−1^ to 3434 cm^−1^, the C–H band at 1427 cm^−1^ intensified, and the bands at 624 cm^−1^ and 590 cm^−1^ merged into a single band at 631 cm^−1^ after reaction, suggesting changes in bonding due to As interaction with Fe/Mn. For FM4, the O–H band shifted from 3431 cm^−1^ to 3439 cm^−1^, the C–H band shifted from 1427 cm^−1^ to 1418 cm^−1^, and the bands at 871 cm^−1^ and 715 cm^−1^ weakened after adsorption.

In summary, the shifts and intensity changes in the O–H bands around 3430 cm^−1^ across all minerals suggest the participation of surface hydroxyl groups in the adsorption process. Changes in the bands near 1400 cm^−1^ indicate potential complexation between surface oxygen-containing functional groups and As(V). Most importantly, the appearance and increased intensity of the As–O–M bands in the 700–900 cm^−1^ region after reaction strongly suggests for the formation of stable inner-sphere complexes between As(V) and Fe/Mn–O groups on the mineral surfaces. These results collectively indicate that As(V) removal by natural manganese minerals is primarily achieved through complexation with hydroxyl groups and metal-oxygen groups on the metal oxide surfaces [72,73].

#### 3.6.3. SEM-EDS Analysis

**Figure 14:**
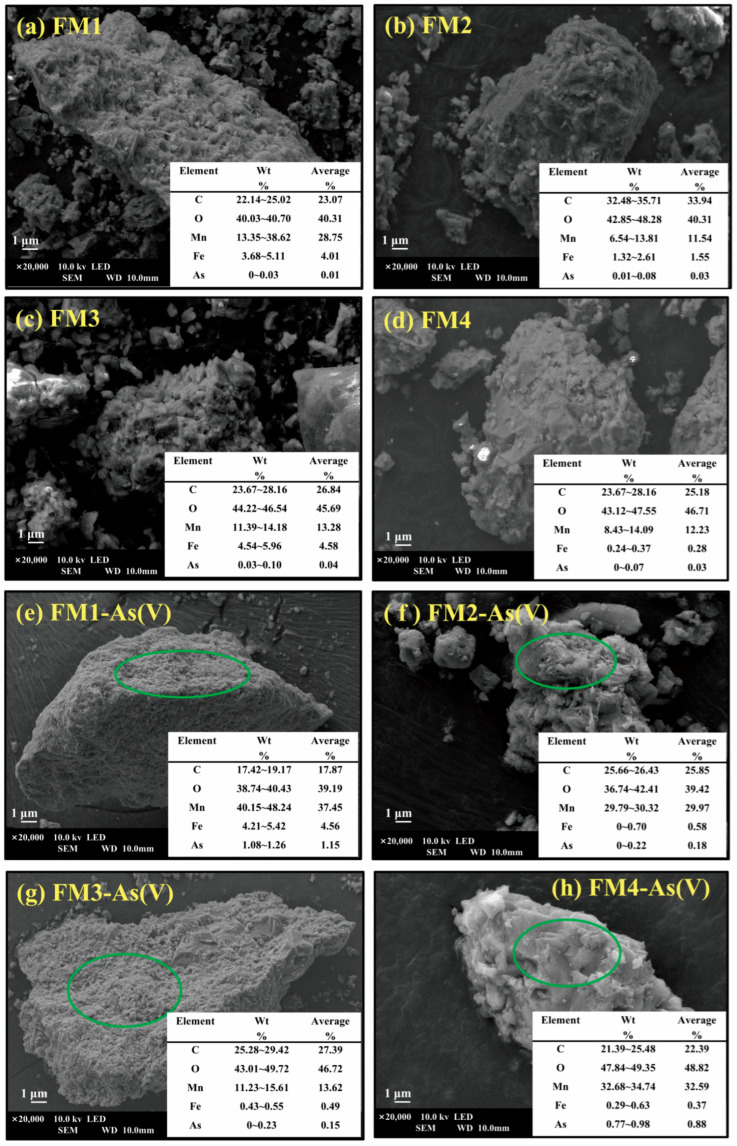
SEM-EDS images of different adsorbents before and after As(V) adsorption. The green circles indicate the regions selected for EDS analysis.

Surface morphology and elemental composition of the minerals before and after reaction were analyzed using SEM-EDS (Figure 14). Pristine FM1 exhibited a porous and finely textured surface (Figure 14a). After As(V) adsorption (Figure 14e), the surface appeared denser. EDS analysis showed a significant increase in average As content (from 0.01% to 1.15%) and Mn content (from 28.75% to 37.45%), while C content decreased (23.07% to 17.87%) and Fe content showed a slight increase (4.01% to 4.56%). These results support surface association of As after reaction and suggest that Mn/Fe-bearing phases may have been involved in the adsorption process; however, the EDS percentages should be interpreted as local comparative information rather than direct proof of a specific active phase or mechanism. Pristine FM2 showed a porous or aggregated granular structure (Figure 14b). After reaction (Figure 14f), the surface became more fragmented. As content increased slightly (0.03% to 0.18%), Mn content increased markedly (11.54% to 29.97%), while Fe (1.55% to 0.58%) and C (33.94% to 25.85%) decreased, resulting in an increased Mn/Fe ratio. Pristine FM3 exhibited a mixed granular and blocky structure (Figure 14c). After adsorption (Figure 14g), the surface consisted of dense granular aggregates. As content increased (0.04% to 0.15%), while Fe content decreased substantially (4.58% to 0.49%), and Mn content remained relatively unchanged, possibly due to Fe dissolution during the process. Pristine FM4 displayed a stacked blocky and granular morphology (Figure 14d). Following As(V) adsorption (Figure 14h), the surface became smoother with fewer visible particles. As content increased significantly (0.03% to 0.88%), Mn content increased sharply (12.23% to 32.59%), and Fe content showed a minor increase (0.28% to 0.37%), suggesting possible involvement of Mn-bearing domains. Taken together, these observations indicate that As became associated with the reacted mineral surfaces, while the concurrent changes in Mn- and Fe-related signals imply that metal-bearing surface domains were involved. Across all four minerals, the increase in the detected As signal after reaction confirms surface enrichment of As, and the associated changes in Mn- and Fe-related signals suggest that Fe/Mn-bearing phases were involved in the immobilization process. Nevertheless, SEM-EDS alone cannot establish whether Mn was the dominant active phase, nor can it by itself demonstrate inner-sphere complexation or surface precipitate formation. These mechanistic aspects should be interpreted together with the FTIR and XPS results [74,75].

#### 3.6.4. XPS

**Figure 15:**
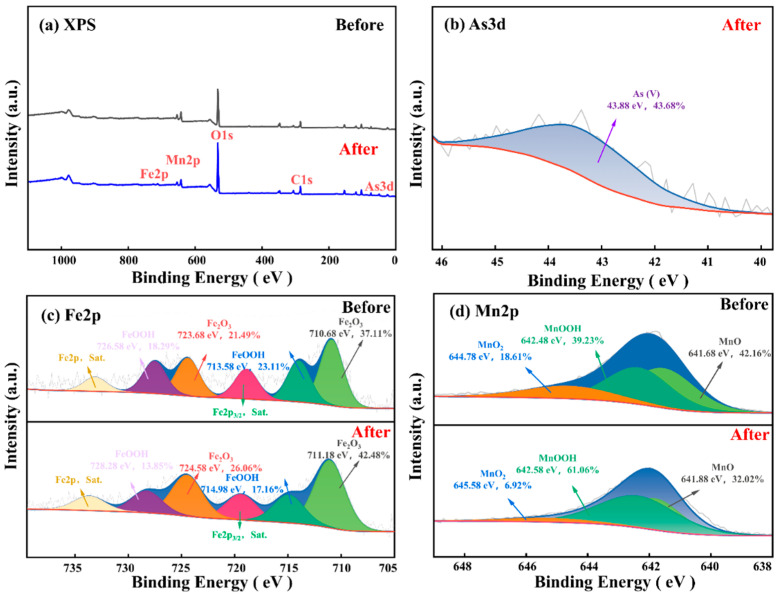
XPS spectra of the solid-phase media of FM1 before and after As(V) adsorption. The red line corresponds to the overall fitted curve, the blue shaded areas are added for visual highlighting and do not represent additional data.

The batch and column experiments demonstrated that FM1 possesses the highest adsorption capacity and the strongest retardation effect for As(V) among the four minerals. To further examine the surface processes associated with superior performance, FM1 was selected as a representative sample for XPS analysis to examine changes in surface elemental composition and chemical states before and after As(V) adsorption (Figure 15).

The survey scan of the reacted FM1 (Figure 15a) shows peaks corresponding to Fe2p, Mn2p, O1s, C1s, and a new peak for As3d, confirming the accumulation of arsenic on the mineral surface. The high-resolution As3d spectrum (Figure 15b) reveals that the adsorbed arsenic is exclusively in the pentavalent state As(V), with no detectable As(III). Together with the Fe and Mn spectral changes discussed below, this result suggests that the adsorption process was dominated by surface-state variation and surface retention of As(V), rather than by an obvious redox transformation of arsenic. As(V) was mainly retained through surface complexation rather than redox-driven precipitation. Such behavior is consistent with previous studies showing that arsenate is commonly immobilized on Fe- and Mn-containing oxides via inner-sphere complexation with surface hydroxyl groups [76].

The Fe2p spectrum (Figure 15c) was deconvoluted into components corresponding to FeOOH and Fe_2_O_3_. After the reaction, the proportion of FeOOH decreased from 41.4% to 31.01%, while Fe_2_O_3_ increased from 58.6% to 68.99%, indicating a transformation in iron phases during adsorption. The Mn2p spectrum (Figure 15d) was fitted with peaks corresponding to MnO_2_, MnOOH, and MnO. Notably, after As(V) adsorption, the proportion of MnO_2_ decreased sharply from 18.69% to 6.92%, MnO decreased from 42.16% to 32.02%, while MnOOH increased substantially from 39.23% to 61.06%. Recent studies have shown that when Mn(II) and Mn(IV) species coexist, MnOOH can form through Mn(II)-induced transformation pathways [77]. The increase in MnOOH indicates the generation of additional active sites for As(V) binding. The appearance of the As(V) signal and the concurrent enhancement of the O1s signal further confirm the involvement of surface oxygen-containing groups in the adsorption process. While Fe phases did undergo some transformation, the changes were less pronounced than those observed for Mn, suggesting that Fe provides a limited number of surface sites for As adsorption. In contrast, the substantial conversion of Mn(IV) and Mn(II) to hydroxyl-rich Mn(III) phases creates favorable conditions for the robust immobilization of As(V) [78,79].

### 3.7. Mechanism Analysis

**Figure 16:**
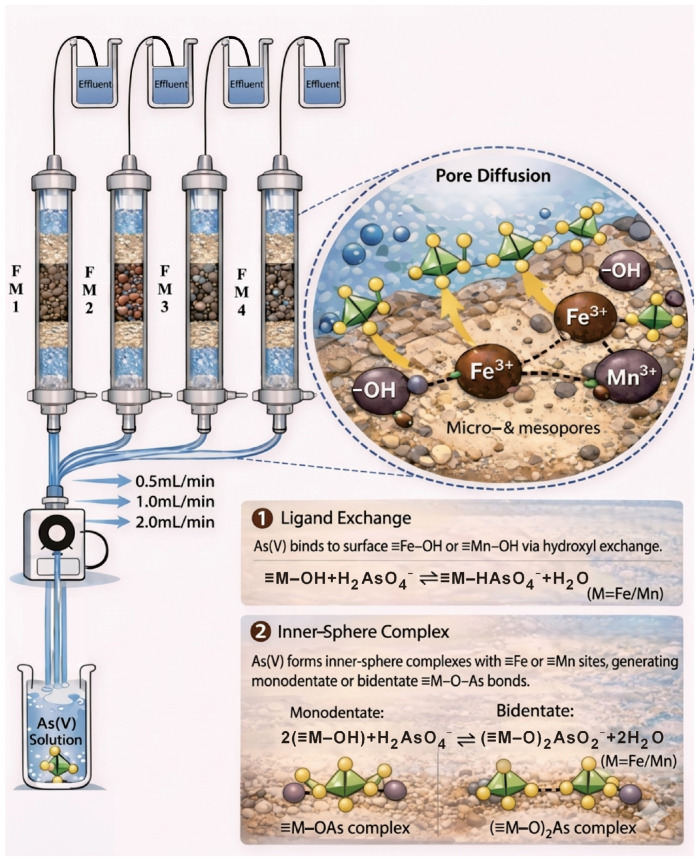
Schematic Diagram of the Mechanism for As(V) Adsorption by Natural Manganese minerals.

Based on the integrated findings from batch and column experiments, modeling, effluent chemistry, and mineral characterization, a conceptual model for the differential As(V) retardation capacities of the natural iron-bearing manganese minerals is proposed (Figure 16). The immobilization of As(V) on the mineral surface can be described schematically as a ligand-exchange process that leads to the formation of stable inner-sphere complexes. In this process, aqueous arsenate species (H_2_${AsO}_{4}^{-}$/${HAsO}_{4}^{2-}$) replace surface hydroxyl groups (≡Fe–OH or ≡Mn–OH) and form direct M–O–As linkages. Such ligand-exchange/inner-sphere complexation mechanisms have been widely reported for arsenate adsorption on Fe- and Mn-containing oxides and oxyhydroxides. Studies have shown that arsenate is commonly retained on these mineral surfaces through direct M–O–As bonding involving surface hydroxyl groups, with mono- and bidentate configurations both being possible depending on surface structure and loading conditions [14,79]. This process significantly enhances the stability of arsenic immobilization. Under the present experimental conditions, this retention behavior was expressed mainly as surface complexation and surface-state variation, rather than as a pronounced redox-driven process. The proposed mechanism is substantiated by the observed increase and shifts in the intensity of As–O–M bands in the FTIR spectra and the slight attenuation of XRD peak intensities after adsorption, confirming the strong interaction between As(V) and the mineral surface. The adsorption process can be represented schematically by the following reactions [60]:

(13) $$\equiv Mn/Fe-OH+H_{2}{AsO}_{4}^{-}⇌\equiv Mn/Fe-HAsO_{4}^{-}+H_{2}O$$

(14) $$2(\equiv Mn/Fe-OH)+H_{2}{AsO}_{4}^{-}⇌(\equiv Mn/Fe-O{)}_{2}AsO_{2}^{-}+2H_{2}O$$

## 4. Conclusions

This study systematically investigated the interactions between natural iron-bearing manganese minerals and As(V) to elucidate the underlying mechanisms, develop a quantitative model, and evaluate their As(V) removal and retardation behavior under the tested continuous-flow conditions. A comprehensive approach was employed, integrating analyses of water chemistry release dynamics, breakthrough kinetics, reactive transport modeling, and multi-scale mineral characterization. The adsorption behavior and hydrochemical responses of four natural manganese minerals (FM1–FM4) with varying Mn/Fe ratios were compared. The key findings are as follows:

(1) Batch experiments revealed that As(V) adsorption on the FM1-FM3 was better described by the Freundlich isotherm model, whereas FM4 was better fitted by the Langmuir model. The pseudo-second-order kinetic model provided equal or better fits overall, especially for FM2–FM4, and was also slightly better than the pseudo-first-order model for FM1. These results suggest that As(V) uptake involved heterogeneous surface adsorption with a strong chemisorption component. Arsenic removal efficiency decreased significantly with increasing initial pH, and FM1 exhibited the highest adsorption capacity among the four minerals (0.066 mmol·g^−1^).

(2) Column experiments demonstrated rapid As(V) retention by all four minerals. For the FM1-packed column, breakthrough (*C*_e_/*C*_0_ ≥ 0.9) occurred at PV 261.1, 272.1, and 136.8 at flow rates of 0.5, 1.0, and 2.0 mL·min^−1^, respectively, and FM1 exhibited the most pronounced tailing among the four minerals. Among the minerals, FM1 showed the strongest retardation and likely formed relatively stable inner-sphere complexation, resulting in strong and less reversible arsenic binding. The combined results from batch and column experiments indicate that chemisorption, primary through surface complexation, is the primary mechanism for As(V) removal by natural manganese minerals. Given the pH_PZC_ values, electrostatic interactions likely played a secondary role under circumneutral to weakly alkaline groundwater conditions. Instead, As(V) immobilization was dominated by surface complexation involving surface hydroxyl and Fe/Mn–O groups, together with changes in Fe/Mn-bearing surface species under the present experimental conditions. Both flow rate and mineral type significantly influence retardation and thus control arsenic transport in groundwater.

(3) The Two-Site Kinetic Attachment Model (TSKAM) successfully simulated the dynamic adsorption of As(V) onto the four minerals at different flow rates, with high goodness-of-fit (overall R^2^ > 0.90, RMSE < 0.05). The excellent applicability of TSKAM confirms the heterogeneous nature of the adsorption sites on the minerals, where adsorption is jointly controlled by a rapid, reversible site (site 1) and a slower, capacity-limited site (site 2). As flow rate increased, the fitted *S*_*max*2_ values decreased, supporting a reduced contribution of the slow site under higher-flow conditions. (4) Multi-technique characterization showed that the major crystalline phases remained identifiable after adsorption and that As(V) immobilization was mainly associated with surface complexation involving surface hydroxyl groups and Fe/Mn–O moieties. FTIR provided evidence for As–O–M bond formation, SEM-EDS confirmed surface enrichment of As, and XPS on FM1 showed that adsorbed arsenic remained mainly as As(V), accompanied by changes in Fe and especially Mn surface species. These results suggest that Mn/Fe ratio alone does not determine adsorption performance; rather, specific surface area, pore structure, pH_PZC_, and Mn surface-state changes collectively influenced As(V) removal.

## Data Availability

The raw data supporting the conclusions of this article will be made available by the authors on request.
